# Dominant Trees in a Subtropical Forest Respond to Drought Mainly via Adjusting Tissue Soluble Sugar and Proline Content

**DOI:** 10.3389/fpls.2017.00802

**Published:** 2017-05-15

**Authors:** Yuanwen Kuang, Yimin Xu, Lingling Zhang, Enqing Hou, Weijun Shen

**Affiliations:** ^1^Key Laboratory of Vegetation Restoration and Management of Degraded Ecosystems, South China Botanical Garden, Chinese Academy of SciencesGuangzhou, China; ^2^Guangdong Provincial Key Laboratory of Applied Botany, South China Botanical Garden, Chinese Academy of SciencesGuangzhou, China; ^3^College of Life Sciences, University of Chinese Academy of SciencesBeijing, China

**Keywords:** functional traits, morphological traits, nutritional traits, subtropical forest, throughfall exclusion, precipitation change

## Abstract

It is well-known that drought has considerable effects on plant traits from leaf to ecosystem scales; however, little is known about the relative contributions of various traits within or between tree species in determining the plant’s sensitivity or the tolerance to drought under field conditions. We conducted a field throughfall exclusion experiment to simulate short-term drought (∼67% throughfall exclusion during the dry season from October to March) and prolonged drought (∼67% throughfall exclusion prolonging the dry season from October to May) and to understand the effects of drought on two dominant tree species (*Michelia macclurei* and *Schima superba*) in subtropical forests of southern China. The morphological, physiological, and nutritional responses of the two species to the two types of drought were determined. There were significantly different morphological (leaf max length, max width, leaf mass per area), physiological (leaf proline) and nutritional (P, S, N, K, Ca, Mg) responses by *M. macclurei* and *S. superba* to prolonged drought. Comparison between the drought treatments for each species indicated that the trees responded species–specifically to the short-term and prolonged drought, with *S. superba* exhibiting larger plasticity and higher adaption than *M. macclurei. M. macclurei* responded more sensitively to prolonged drought in terms of morphology, proline content, and nutritional traits and to short-term drought with regard to soluble sugars content. The differential species-specific responses to drought will allow us to estimate the changes in dominant trees in subtropical forests of China that have experienced a decade’s worth of annual seasonal drought.

## Introduction

Both global climate models (Fischer et al., 2013; Singh et al., 2013) and observed precipitation trends (Marvel and Bonfils, 2013) have revealed that climate change is intensifying the hydrologic cycle and is expected to increase the variation in precipitation regimes worldwide. The variation in precipitation patterns at both regional and global scales will occur with great spatio–temporal variability and complexity (Beier et al., 2012). At a global scale, the amount of rainfall is predicted to increase at mid– and high–latitudes and to decrease at low latitudes, with notable changes in seasonal intensity and frequency (Greve et al., 2014). Regionally, e.g., in south China, it will be drier in the dry season and wetter in the wet season with ongoing climate change (Zhou et al., 2013). Accordingly, the dry season has lasted longer and the wet season has had more intensive rainstorms in the last 10 years, despite the total annual precipitation amount not changing significantly, in some regions of south China (Zhou et al., 2011).

Changes in precipitation regimes have considerable ecological and evolutionary impacts on terrestrial ecosystems (Pratt and Mooney, 2013; Moran et al., 2014; Zeppel et al., 2014; Knapp et al., 2015). Physiological properties of plant species (Coe and Sparks, 2014); encroachment of woody plants (Kulmatiski and Beard, 2013); tree mortality (Anderegg et al., 2015); forest structure (Hoeppner and Dukes, 2012); evapotranspiration dynamics (Raz-Yaseef et al., 2012); plant community composition (Cleland et al., 2013); biomass and net primary productivity (Hovenden et al., 2014; Wilcox et al., 2015); litterfall (Travers and Eldridge, 2013); the availability, uptake, transport, and accumulation of plant nutrients (including N, P, S, K, Ca, and Mg) (Rouphael et al., 2012); soil respiration (Thomey et al., 2011); and soil microbial communities (Zeglin et al., 2013), have been well documented to be affected by the rainfall patterns.

Plant functional traits, e.g., morphological, bio-physiological, and nutritional traits of leaves and roots—can individually or in combination, indicate the responses of plants to environmental changes (Wright et al., 2005; Meng et al., 2009; Pratt and Mooney, 2013), and further provide insights into the adaptation strategies and survival abilities of plants (Picotte et al., 2009). Droughts have considerable effects on plant functional traits from leaf to ecosystem scales (Moreno–Gutiérrez et al., 2012; Flanagan and Farquhar, 2014). However, the drought responses of dominant species in tropical and subtropical forests to drought remains relatively poorly understood (Beier et al., 2012), since knowledge of direct and indirect effects of droughts has usually been derived from studies of pot experiments with seedlings, whose results are generally inconsistent with field manipulations of mature trees (Liu et al., 2011). Pot experiments may have hindered the understanding of drought responses among species. Throughfall exclusion experiments (TEE) and throughfall displacement experiments (TDE) in forest ecosystems (Borken et al., 2006; Nepstad et al., 2007; Gaul et al., 2008; da Costa et al., 2010; Limousin et al., 2010) provided better methods of determining whether the morphological, physiological and nutritional changes occur simultaneously or consistently between species or in response to different types of drought.

*Michelia macclurei* and *Schima superba* are two dominant angiosperm species in subtropical evergreen broadleaf forests in southern China. Both the species are evergreen tall arbors with important ecological and commercial traits. However, *M. macclurei* is distinguished from *S. superba* by its rooting systems. *M. macclurei* is characterized by shallow lateral roots (Liao et al., 1995) and large proportion of fine roots in the total roots biomass (Li et al., 1993), whereas *S. superba* has deep tap roots and a lower proportion of fine roots (Zheng, 2006). The species’ distinct root systems facilitates the study of the potential differences in the species’ responses to two types of seasonal drought (short-term and prolonged drought).

In this study, we designed a TEE experiment in a subtropical evergreen broadleaf forest, the most common forest type and the largest carbon sink in China (Piao et al., 2009), to investigate the morphological, physiological, and nutritional responses of *M.* and *S. superba* to different seasonal droughts. We hypothesized that there would be differences between the two species in their responses to the different types of droughts. Our particular interest was whether similar adaptive strategies would emerge, based on the variation in morphological, physiological, and nutritional traits. We expected to find evidence of differential responses to drought, since consistent differences in responses to drought among tree species would likely indicate compositional shifts in forests (da Costa et al., 2010). This type of research will help to estimate the possible changes that may have occurred in the dominant trees in subtropical forests of China as a result of seasonal drought that have occurred over the last decade (Zhou et al., 2013) and also to future drought.

## Materials and Methods

### Experimental Site

The experimental site is located 80 km southwest of Guangzhou (southern China) in the Heshan National Field Research Station Forest Ecosystem (112°54′E, 22°41′N, 80 m a.s.l.). The area has a subtropical climate with a mean annual temperature of 21.7°C, with the coldest month being January (13.1°C) and the hottest month, July (28.7°C). The mean annual precipitation is 1700 mm with a range of 1060–2060 mm recorded during the past 4 decades. The dry season, lasting from October to March, is distinct from the wet season, which lasts from April to September and is when the majority of the rainfall occurs. In the mid-1980s, researchers constructed an experimental plantation forest mixed with native broadleaf species at this station. After 30 years of succession, the plantation is currently characterized as secondary evergreen broadleaf forest dominated by *S. superba* and *M. macclurei* in the canopy layer; by *Ilex asprella*, *Psychotria asiatica*, and *Melicope pteleifolia* in the shrub layer; and by *Lophatherum gracile* and *Adiantum flabellulatum* in the herb layer. The mean canopy height and the diameter at breast height of this forest are approximately 14 m and 22 cm, respectively. The density of the trees was 1660 stem⋅ha^-1^ in 2012 (Jiao and Shen, 2014). The soil is laterite developed from sandshale. The average slope of this forest is approximately 15°.

### Experimental Design

Based on the decreasing trend of rainfall in the dry season in this region (Zhou et al., 2011), we designed a TEE in this secondary evergreen broadleaf forest. Throughfall exclusion was achieved by using a system of plastic panels and plastic-lined guttering installed at a height of 1.5 m above the ground (Fisher et al., 2007). Eight 12 m × 12 m plots (at least 2 m away from each other) randomly distributed in the forest were subjected to one of two treatments reflecting two types of seasonal drought via throughfall exclusion: (1) drier dry season (DD) treatment, a relatively short-term drought and (2) extended dry season (ED) treatment, a prolonged drought. Another four plots were not subjected to drought treatment, reflecting the influence of trenching control (TC).

Briefly, in the DD treatment, in the dry season (from October to March), net input throughfall was reduced by ∼67% compared with the TC treatment. In the ED treatment, ∼67% of net input throughfall was again excluded, and the exclusion was continued until May using the same system; this treatment simulated a prolonged dry season from October to May. In both the DD and ED treatments, trenches (60–80 cm deep to bedrock) were dug and PVC panels were embedded around the perimeters of the plots to reduce the lateral inflow of water through the soil matrix and surface runoff from outside the plots. In the TC treatment, no throughfall exclusion facility was constructed, but trenches were used as a control for the DD and ED treatments. The effect of rainfall exclusion could be detected by comparing the DD and ED treatments to the TC treatment.

### Field Sampling

Prior to the drought treatment, the plots were inventoried and trees selected for sampling were tabbed with red plastic tape. The total number of sample *S. superba* was 37 (DD 12, ED 12, TC 13), and of *M. macclurei*, 31 (DD 11, ED 10, TC 10). To diminish the influence of season on the morphological, physiological, and nutritional traits of the two species, the trees were sampled at different times according to the treatments: (1) background sampling (September, 2013) for all treatments, (2) the end of the DD in comparison with the TC treatment (March, 2014), (3) the end of the ED in comparison with the TC treatments (May, 2014). At each sampling, at least 20 mature leaves were collected from the canopy of each tabbed trees in each treatment. To minimize the influence of leaf age on the traits, the leaves were sampled from among those developed in the previous growing season (April–June, 2013). Approximately 10 g (fresh weight) fine roots were collected from below the surface soils of each tabbed tree. The leaves and the fine roots were stored in ice bags, and immediately carried to the laboratory for analysis.

### Laboratory Analysis

#### Leaf Morphology

The sampled leaves were divided into two groups: one for morphological and nutritional measurement, the other for measurement of chlorophyll and other physiological traits. The maximum length (L, mm), maximum width (W, mm), and area (A, mm^2^) of each leaf were determined with a portable area meter (Li-3000C, Li-Cor Bioscience, UAS). The leaves were then oven-dried at 60°C for at least 48 h and weighed for dry mass. The leaf mass per area (LMA, g⋅mm^-2^) and the ratios of maximum length to maximum width (L/W) were calculated.

#### Leaf Physiological Traits

Frozen leaves (approximately 0.2 g each) from individual trees were cut and dipped into 5 mL 80% acetone to determine chlorophyll a and chlorophyll b (Chl_a_ and Chl_b_, respectively) contents. The contents of Chl_a_ and Chl_b_ (ug⋅mg^-1^, fresh weight) were determined by the absorption at 663 and 645 nm, respectively, using a spectrophotometer (Unico, Shanghai, China). Chl_a_ and Chl_b_ were summed to arrive at the total chlorophyll (Chl_Total_).

The contents of soluble sugar in the leaves and fine roots of individual trees were determined according to the methods described in Kuang et al. (2012). Briefly, approximately 50 mg of the oven-dried leaf or fine root powder was extracted with 80% ethanol (v/v) at 85°C for 1 h. The solution was then centrifuged at 12,000 r for 10 min. The resulting supernatants were then combined, treated with activated charcoal, and evaporated to dryness in a vacuum evaporator. The residues were then re-dissolved in distilled water, then subjected to soluble sugar analysis using the anthrone–sulfuric acid method.

Free proline was extracted and determined as described by Hamid et al. (2003). Leaf and fine root samples were extracted with 3% sulphosalicylic acid. Extracts were mixed with 2 mL glacial acetic acid and 2 mL acid ninhydrin and maintained for 1 h at 100°C. Cold toluene was then added and the samples were shaken. Absorbance was read at 520 nm. The amount of proline was determined from a standard curve.

#### Nutritional Analysis

The powdered leaves were digested with concentrated nitric and perchloric acid (8:1, *v*/*v*) in a microwave system (Multiwave 3000, Anton Paar, Austria). The analytical procedure was performed using the method established by Dong (1996). After digestion, the concentrations (mg⋅kg^-1^) of the nutrients potassium (K), calcium (Ca), magnesium (Mg), and phosphorus (P) were determined using an inductively coupled plasma atomic emission spectrometer (ICP–OES, Optima 2000, USA). The content of nitrogen (N) was measured using an elemental analyzer (Flash EA 1112 Series, Thermo Finnigan, Milan, Italy). The content of sulfur (S) was determined using a sulfur analyzer (LTDL–9, China). The reliabilities of the analytical procedure were tested with both blanks and national standard reference materials (GSV–3).

### Statistical Analysis

A linear mixed effects regression was performed by using Rstudio software (v0.98.953^1^; R package, lme4), where the species and the treatment were considered as fixed factors, and individuals nested in plots as random factors, to analyze the effects of drought treatment, the species, and their interactions on the morphological, physiological, and nutritional traits. Mean comparison was performed using ANOVAs between the drought and TC treatments to test whether the drought had significantly influenced the leaf (fine roots) traits. Effects with probabilities of *p* < 0.05 were considered to be significant. The data are shown as the mean ± standard deviation (SD).

## Results

### Leaf Traits

**FIGURE 1:**
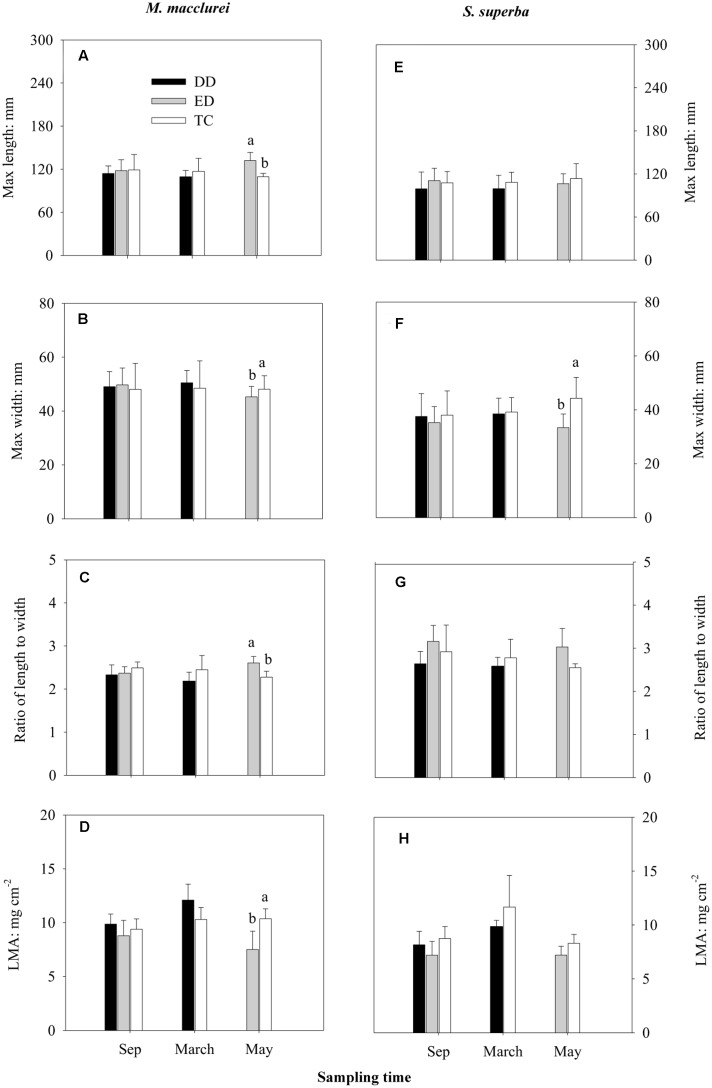
**Effects of drier dry season drought (DD), prolonged drought (ED), and trenching control (TC) on the leaf max length** **(A,E)**, max width **(B,F)**, the ratios of max length to max width **(C,G)**, and the leaf mass per area (LMA; **D,H**) of *M. macclurei* (left column) and *S. superba* (right column). Data are shown as the mean and one standard deviation. Different letters show the significant differences among the treatments (*p* < 0.05).

Prior to the throughfall exclusion, there were no significant differences in the leaf traits (L, W, L/W, and LMA) among the treatments in either *S. superba* or *M. macclurei* (**Figure 1**). For *M. macclurei*, the prolonged drought (ED) significantly influenced the leaf traits, i.e., it significantly increased L and L/W and decreased W and LMA in comparison to the TC treatment. However, the short-term drought (DD) had no significant effects on the leaf traits of *M. macclurei*. For *S. superba*, the two types of drought had no significant effects on morphological traits except that the ED treatment notably decreased the leaf width. However, there were significant differences between the two species in the L and W response to the ED treatment. Leaf mass area responded significantly differently to both ED and DD treatments due to the interaction of species and treatment (*p* < 0.05, **Table 1**).

**Table 1:** Effect of drought treatment, species, and their interactions on the morphological, physiological, and nutritional traits.

Tissue	Index	Drier dry season (DD treatment)						Extended dry season (ED treatment)					
		
		Species		Treatment		Species × Treatment		Species		Treatment		Species × Treatment	
							
		*F*	*p*	*F*	*p*	*F*	*p*	*F*	*p*	*F*	*p*	*F*	*p*
Leaves	L	3.589	0.066	2.656	0.112	0.018	0.892	2.653	0.117	2.545	0.126	10.833	**<0.05**
	W	24.261	**<0.001**	0.067	0.796	0.331	0.570	17.238	**<0.001**	14.309	**<0.001**	4.392	**<0.05**
	L/W	13.668	**<0.001**	6.340	**<0.05**	0.174	0.679	22.499	**<0.001**	30.266	**<0.001**	2.586	0.120
	LMA	1.332	0.257	0.001	0.977	5.942	**<0.05**	8.933	**<0.01**	27.668	**<0.001**	4.619	**<0.05**
	Chl_a_	4.831	**<0.05**	0.284	0.597	1.782	0.192	2.412	0.130	7.957	**<0.01**	1.076	0.308
	Chl_b_	0.361	0.552	0.056	0.815	2.152	0.152	2.309	0.138	7.547	**<0.05**	1.278	0.267
	Chl_Total_	3.045	0.091	0.215	0.646	2.026	0.165	2.523	0.122	8.277	**<0.01**	1.203	0.282
	Soluble sugar	0.962	0.354	0.134	0.723	2.377	0.159	4.554	0.061	5.333	**<0.05**	0.960	0.352
	Proline	0.073	0.792	1.443	0.257	1.756	0.215	82.816	**<0.001**	75.373	**<0.001**	54.950	**<0.001**
	N	0.765	0.394	1.098	0.310	0.532	0.476	8.127	**<0.05**	9.591	**<0.001**	1.352	0.262
	P	11.051	**<0.05**	0.530	0.471	0.008	0.927	10.046	**<0.05**	8.086	**<0.05**	5.965	**<0.05**
	S	0.277	0x.602	0.719	0.403	2.487	0.125	2.960	0.093	0.020	0.887	9.345	**<0.01**
	K	8.186	**<0.01**	1.866	0.181	4.670	**<0.05**	11.778	**<0.001**	11.991	**<0.001**	17.800	**<0.001**
	Ca	3.292	0.087	0.197	0.661	0.157	0.695	7.814	**<0.01**	0.144	0.706	12.694	**<0.01**
	Mg	32.927	**<0.001**	0.001	0.974	0.168	0.685	198.184	**<0.001**	1.641	0.211	18.051	**<0.001**
Fine roots	Soluble sugar	25.531	**<0.001**	0.758	0.408	0.935	0.360	1.051	0.330	3.128	0.106	0.144	0.712
	Proline	22.978	**<0.001**	0.991	0.342	0.900	0.365	121.166	**<0.001**	26.044	**<0.001**	1.605	0.236


*The analysis was based on a linear mixed model fitted by maximum likelihood with the species and the treatment as fixed factors, and individuals nested in plots as random factors. The significance was set at *p* < 0.05 levels. The number bolded means the effect is significant.*

### Physiological Traits

#### Leaf Chlorophyll

**FIGURE 2:**
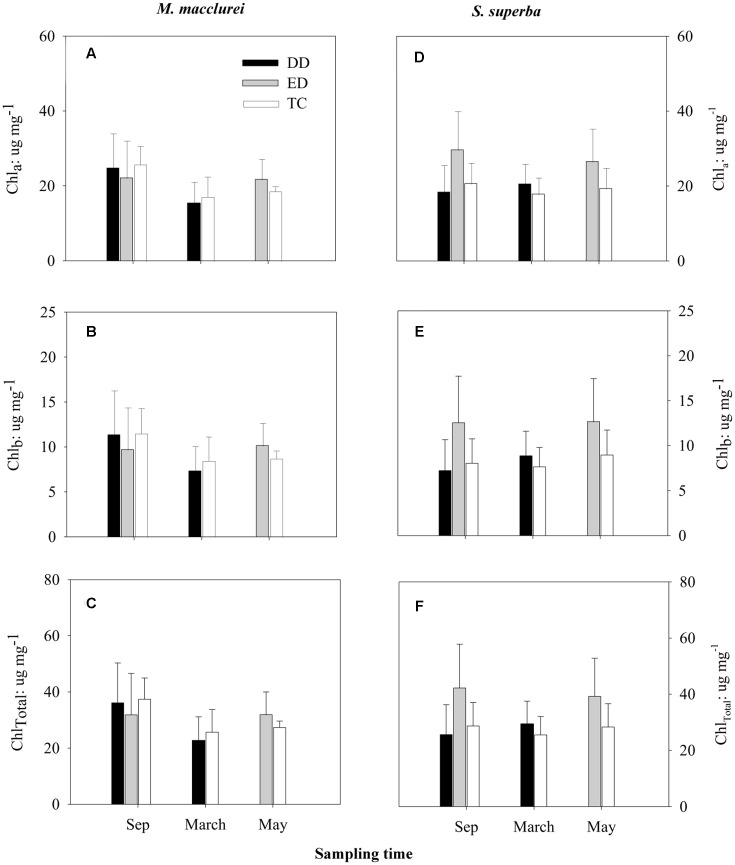
**Responses of leaf chlorophyll a (Chl_a_;** **A,D)**, chlorophyll b (Chl_b_; **B,E**), and total chlorophyll (Chl_Total_; **C,F**) to the drier dry season drought (DD), prolonged drought (ED), and trenching control (TC) of *M. macclurei* (left column) and *S. superba* (right column). Data are shown as the mean and one standard deviation.

Similar to the leaf morphological traits, leaf chlorophyll contents showed no significant differences among the treatments prior to the drought (Sep 2013). Unexpectedly, neither the DD (March 2014) nor the ED (May 2014) treatments significantly influenced the contents of Chl_a_, Chl_b_, or Chl_Total_ in the leaves of either species, although Chl_a_, Chl_b_, and Chl_Total_ showed decreasing tendencies in DD treatment and increasing tendencies in ED treatment for *M. macclurei* and increasing tendencies in both DD and ED treatments for *S. superba*. Trenching had little effect on leaf chlorophyll contents (**Figure 2**). The species and treatment interaction was not a significant factor in the response of leaf chlorophyll to drought (*p* = 0.192, 0.152, 0.165, respectively, in DD treatment, and *p* = 0.308, 0.267, 0.282, respectively, in ED treatment, **Table 1**).

#### Soluble Sugars and Proline

**FIGURE 3:**
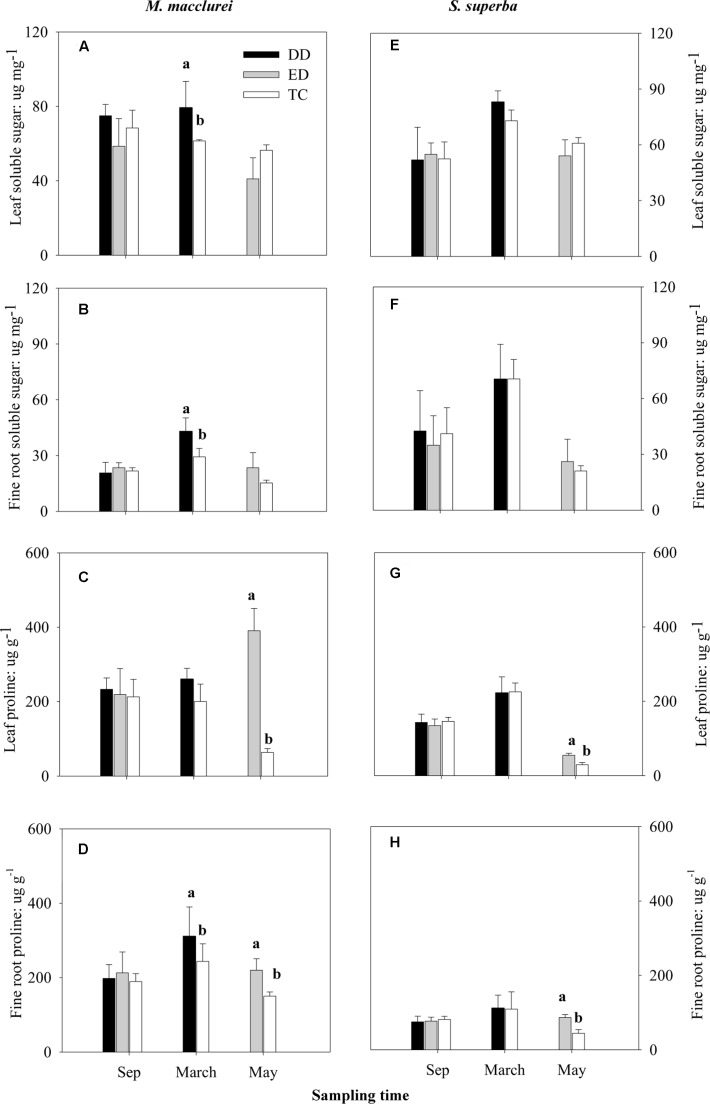
**Responses of soluble sugar and proline in** **(A,C,E,G)** leaves and **(B,D,F,H)** fine roots of *M. macclurei* (left column) and *S. superba* (right column) to the drier dry season drought (DD), prolonged drought (ED), and trenching control (TC) treatments. Data are shown as the mean and one standard deviation. Different letters denote a significant difference among the treatments (*p* < 0.05).

Prior to the drought (September, 2013), the osmotic solutes (soluble sugars and proline) in both the leaves and fine roots of *M. macclurei* and *S. superba* did not differ significantly among the treatments. The DD treatment significantly increased the soluble sugar content in the leaves and fine roots of both *M. macclurei* (**Figures 3A,B**) and *S. superba* (**Figure 3F**). Meanwhile, the DD treatment caused a remarkable increase in proline content in the fine roots of *M. macclurei* (**Figure 3D**). The proline content in the leaves of both *M. macclurei* and *S. superba* did not seem to be influenced by the short-term drought (**Figures 3C,G**). The ED treatment did not notably influence the soluble sugars in the leaves and fine roots of the two species (**Figures 3A,B,E,F**). It did cause significantly higher proline content in the leaves and fine roots of both *M. macclurei* and *S. superba* (**Figures 3C,D,G,H**). Unexpectedly, only the proline content in the leaves responded significantly differently between the species to the extended drought (*p* < 0.001, **Table 1**).

### Nutritional Traits

**FIGURE 4:**
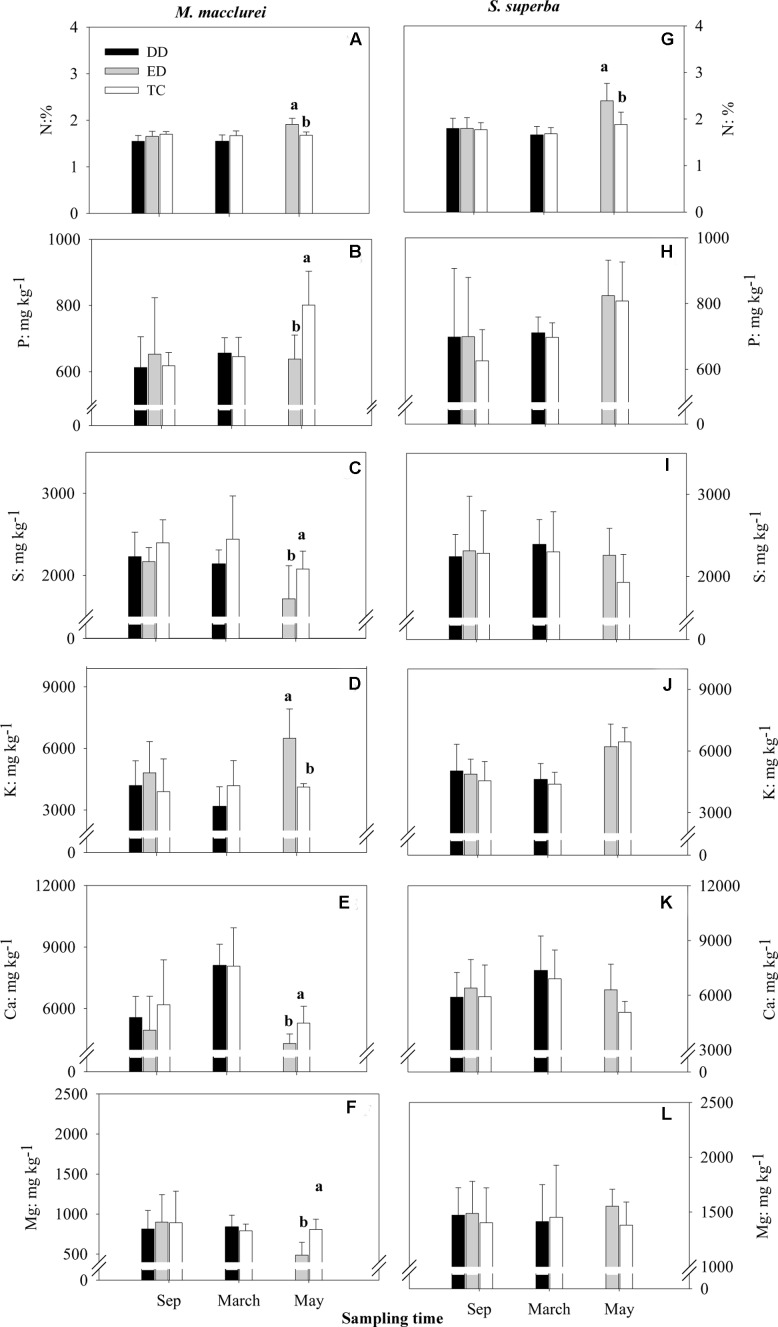
**Concentrations of N** **(A,G)**, P **(B,H)**, S **(C,I)**, K **(D,J)**, Ca **(E,K)**, and Mg **(F,L)** in the leaves of *M. macclurei* (left column) and *S. superba* (right column) responding to the drier dry season drought (DD), prolonged drought (ED), and trenching control (TC) treatments. Different letters denote significant differences among the drought treatments (*p* < 0.05).

None of the nutrients (N, P, S, K, Ca, and Mg) in the leaves were significantly different among treatments prior to the drought treatments but responded to the drought differently in each species (**Figure 4**). For *M. macclurei*, N and K content increased and P, S, Ca, and Mg content decrease significantly in response to the prolonged drought. The short-term drought (DD) treatment and the TC had no notable influence on any of the studied nutrients. For *S. superba*, the nutrients in the leaves were not influenced by either the short-term or the prolonged drought, except for N, which increased significantly in the prolonged drought (**Figures 4G–L**). Interestingly, leaf nutrients except for N responded to the extended drought significantly differently in the two species, while in the short-term drought leaf nutrients except for N had no significantly different responses between the species (**Table 1**).

## Discussion

The two species had significantly different responses to the prolonged drought in leaf L, W, and LMA (*p* < 0.05, **Table 1**), but in the short-term drought, only LMA responded differently (*p* < 0.05, **Table 1**). The responses of leaf morphologies to the two types of drought were species-specific, with *M. macclurei* obviously showing more sensitivity to prolonged drought than short-term drought and with *S. superba* showing more plasticity to drought in leaf morphologies (**Figure 1**). Under prolonged drought conditions, *M. macclurei* attempted to increase the leaf L, decrease the leaf W (**Figures 1A,B**), and narrow the leaf mass per area to adapt to the drought (**Figures 1C,D**). This type of adjustment can enhance the water–use efficiency (Knight and Ackerly, 2003) and reduce water loss via the smaller leaf area (Rozendaal et al., 2006). The significantly increased L/W of *M. macclurei* (**Figure 1C**) implied its higher sensitivity to prolonged drought in comparison to *S. superba.* Since the increased L/W can enlarge the leaf boundary layer, it can reduce respiration and retain leaf lipids for use in physiological activities (Han et al., 2014). Generally, plants with higher drought tolerance have longer and narrower leaves and larger LMA (Niinemets, 2001; Liu et al., 2011). The significantly different leaf morphologies in the prolonged drought treatment implied that *M. macclurei* is more sensitive to drought than *S. superba*, which showed higher plasticity to the significant changes in soil water availability. The preferential variation in leaf morphology may be one of the response mechanisms of *M. macclurei*, along with its large amount of shallow fine roots in comparison to *S. supeerba* (Li et al., 1993; Liao et al., 1995; Zheng, 2006). However, since mature trees respond more slowly to the changes in soil water than do woody seedlings (Liu et al., 2011), it is important to have longer-term monitoring data to gather considerably more evidence before drawing this conclusion.

Leaf chlorophyll can quickly respond to abiotic stress such as drought (Sapeta et al., 2013). However, the response of leaf chlorophyll did not significantly differ between *M. macclurei* and *S. superba* neither in the short drought (*p* = 0.192, 0.152, and 0.165, for Chl_a_, Chl_b_, and Chl_Total_, respectively; **Table 1**) nor the prolonged drought (*p* = 0.308, 0.267, and 0.282; **Table 1**). The insignificant differences may be ascribed to the drought intensity, drought duration, tree species, or other factors. Longer or more intense drought treatments may give further insight into the species’ chlorophyll responses. After all, the drought in this study was a rainfall reduction to one-third of normal, for one more month than the natural dry season.

Osmoregulation *via* accumulation of osmoregulators is recognized as one of the most important tolerance mechanisms that plants develop to adapt to a water deficit (Hoekstra and Buitink, 2001). The results in the present study showed that the responses of leaf proline under prolonged drought significantly differed between the species (*p* < 0.001, **Table 1**), implying a species-specificity in osmotic adjustment. Proline, as the best-known osmoregulator in plants (Ben Ahmed et al., 2009; Hessini et al., 2009; Dobrá et al., 2011), plays a key role in regulating redoxpotential (Shao et al., 2006), scavenging the hydroxyl radical and acting as a cytosolic osmoticum (Kishore et al., 2005), and is thought to protect plant cells by stabilizing membranes (Szabados and Savouré, 2010). The prolonged drought caused *M. macclurei* (more than 6 times the TC, **Figure 3C**) and *S. superba* (∼2 times the TC, **Figure 3G**) to significantly accumulate leaf proline. The result implied that both *M. macclurei* and *S. superba* could actively adjust proline in leaves in response to extended drought, which may be an adaptive strategy to protect it against drought stress and increase drought stress tolerance (Cvikrová, 2013; Königshofer and Löppert, 2015).

Furthermore, we found the short-term drought significantly increased the accumulation of soluble sugars in both leaves and fine roots of *M. macclurei* (**Figures 3A,B**) rather than of *S. superba* (**Figures 3E,F**). Soluble sugars, the main contributors to osmotic adjustment under drought stress (Hessini et al., 2009; Hummel et al., 2010), are important in regulating plant metabolism and development (Wingler and Roitsch, 2008; Bolouri-Moghaddam et al., 2010). The significant accumulations of soluble sugars implied that *M. macclurei* could adaptively respond to short-term drought, which may be a biochemical mechanism helping it to acclimate to drought (Sanders and Arndt, 2012), improve drought tolerance (Lee et al., 2008; Regier et al., 2009), or enhance resistance to drought (Elsheery and Cao, 2008).

Interestingly, *M. macclurei* had remarkable accumulation of proline in fine roots under both types of drought conditions (**Figure 3D**), while *S. superba* had higher proline accumulation only during prolonged drought (**Figure 3H**), further implying that the osmotic responses to drought could be different between species. For *M. macclurei*, the remarkable accumulation of proline in fine roots was relatively higher compared to that in the leaves in the short-term drought (**Figures 3C,D**) indicating that fine roots have a more sensitive osmotic response to drought than can leaves. For *S. superba*, proline was more sensitive to prolonged drought than the soluble sugars were. The significantly higher accumulation of osmotic solutes in the fine roots of *M. macclurei* suggested this species had a higher sensitivity to osmotic adjustment, indicating that it could maintain water absorption more efficiently under drought conditions than *S. superba*.

The significantly different responses of P, S, K, Ca, and Mg (*p* < 0.05, 0.01, 0.001, 0.01, and 0.001, respectively) to prolonged drought and of K (*p* < 0.05) to short-term drought (**Table 1**) between the two species was similar to those of other species (Hessini et al., 2009; Hummel et al., 2010). The significant increase in N content during the prolonged drought in the leaves of both species (**Figures 4A,G**) could be explained by the increase in nitrate content (Kameli and Lösel, 1995) as a result of rapid decline in nitrate reductase activity (Sivaramakrishnan et al., 1988), presumably due to the dieback of the microbial biomass that is induced by drought (Borken and Matzner, 2009). The significantly lower P, S, Ca and Mg content in the leaves of *M. macclurei* during the prolonged drought relative to the other treatments indicated that *M. macclurei* can more easily respond to prolonged rather than short-term drought, since the transpiration rate is the primary determinant of nutrients distribution within plants (de Freitas et al., 2011). The unexpectedly significantly increased K content in the leaves of *M. macclurei* under the ED treatment may have been remobilized from older tissues, which is one mechanism for providing sufficient K to the younger tissues within a plant. It has been reported that the K concentrations may increase even if the drought was of short duration and low intensity (McWilliams, 2003), since the K ion in plant tissues can act as one of the main contributors to osmotic adjustment during drought (Hessini et al., 2009; Hummel et al., 2010).

For *S. superba*, however, the nutrients (except for N) did not vary significantly under the two drought treatments, implying that *S. superba* may be more plastic and have higher adaption to drought relative to *M. macclurei*. However, longer-duration and higher-intensity droughts may provide more evidence for assessing the adaptation of plants to drought.

Results from this study provide new insights into the differentiation of subtropical tree species’ responses to two types of drought *via* adjusting morphological, physiological and nutritional traits. The species-specificity of responses between the trees should be taken into consideration in the development of some models, e.g., global impact models (Fischer et al., 2013; Singh et al., 2013), forest ecosystem water-use efficiency process-based model (Huang et al., 2016), assessing the forest responses to seasonal drought or in the management of tree/forest practice under drought conditions. The inconsistency of species’ responses can help facilitate the future design of through-fall exclusion experiments enabling common and comparable treatments to be imposed in disparate species. This facilitation will allow experiments to more realistically simulate precipitation regimes and more accurately assess the ecological consequences.

## Conclusion

There are significantly different morphological (leaf max length, leaf max width, LMA), physiological (leaf proline) and nutritional (P, S, K, K, Ca, Mg) responses to throughfall–exclusion–induced prolonged drought between *M. macclurei* and *S. superba.* Under both short-term and prolonged drought conditions, *S. superba* exhibited larger plasticity and higher adaption than did *M. macclurei*, based on the variation in morphological, biochemical, osmotic, and nutritional traits in the trees during the drought treatments. *M. macclurei* responded more sensitively to the prolonged drought with respect to morphology, proline content, and nutritional traits and to the short-term drought with respect to soluble sugars content than did *S. superba*. The differential species-specific responses to drought will allow us to estimate the possible changes in dominant trees in subtropical forests of China which have experienced a decade’s worth of annual seasonal drought.

## Author Contributions

WS and YK conceived and designed the experiments. YX and LZ performed the experiments. YX, YK, and EH analyzed the data. YK wrote the manuscript; other authors provided editorial advice.

## Conflict of Interest Statement

The authors declare that the research was conducted in the absence of any commercial or financial relationships that could be construed as a potential conflict of interest. The reviewer SR and handling Editor declared their shared affiliation, and the handling Editor states that the process met the standards of a fair and objective review.

## Footnotes

1 http://www.rstudio.com/
